# Supplementary material for: ABC Transporters in Prorocentrum lima and Their Expression Under Different Environmental Conditions Including Okadaic Acid Production
Source: Mar Drugs. 2019 Apr 30;17(5):259. doi: 10.3390/md17050259 (PMC6563122; doi:10.3390/md17050259)
Supplement: Supplementary file 1 [file marinedrugs-17-00259-s001.pdf]

Supplementary Table 1 Subcellular localization of proteins predicted using CELLO (Yu et al, 2014), targetP v1.1 (Emanuelsson et al., 2000), ProtComp 9.0 and Euk-mPLoc 2.0 (Chou and Chen, 2010)

Gene Name	CELLO	targetP v1.1	ProtComp 9.0	Euk-mPLoc 2.0
ABCB1	PlasmaMembrane	other *	Plasma membrane	Cell membrane
ABCC1	PlasmaMembrane	other *	Plasma membrane	Cell membrane
ABCG2	PlasmaMembrane	other *	Plasma membrane	Cell membrane

* mean the proteins are non-chloroplast, non-mitochondrial and non-secretory protein.

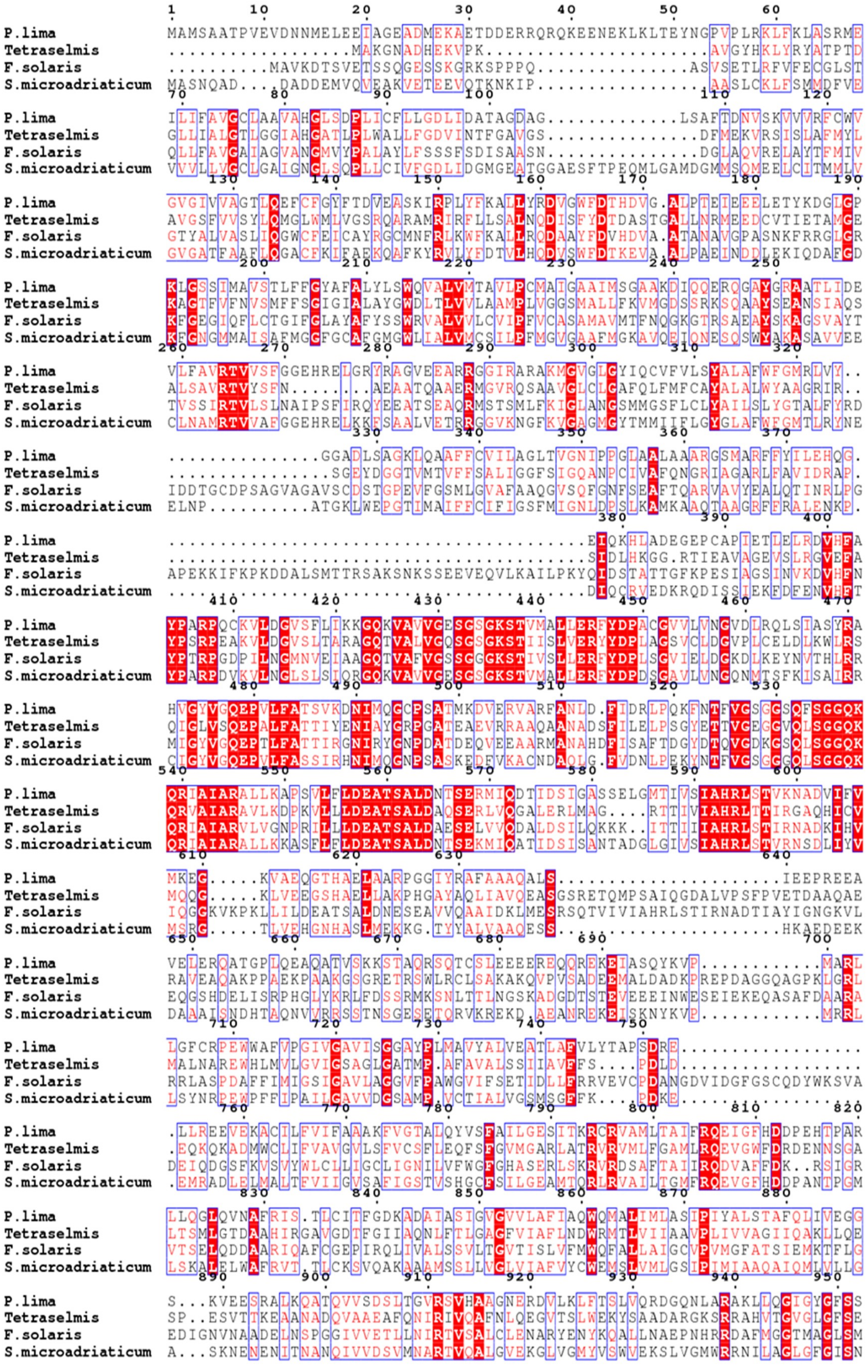
P.lima
Tetraselmis
F.solaris
S.microadriaticum

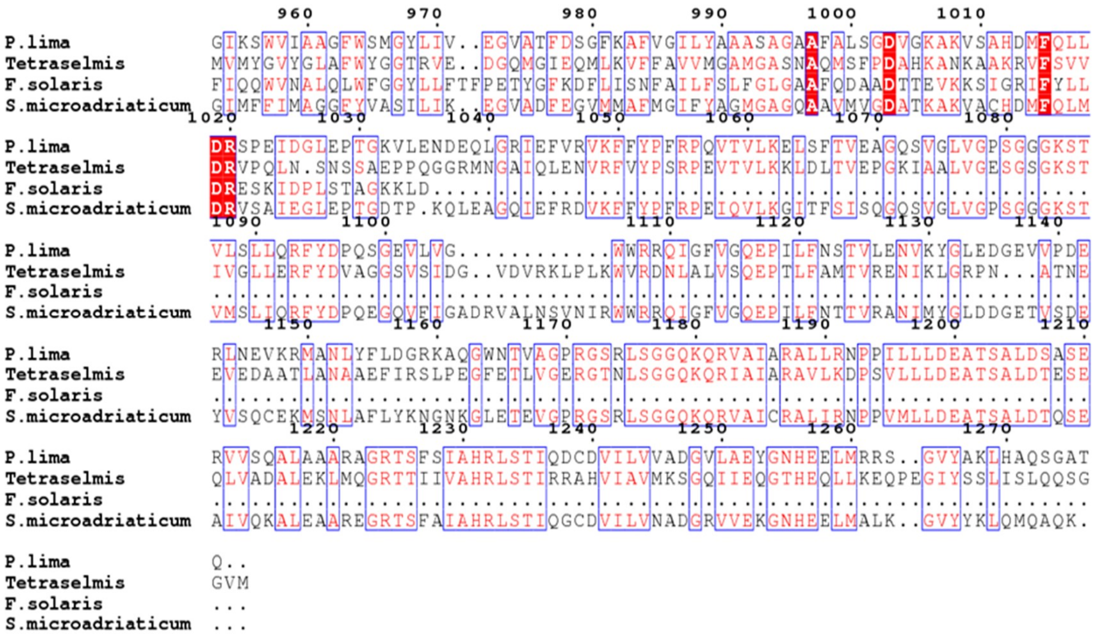

Supplementary Figure 1 Alignment of ABCB1 from *P. lima* with other algae (*Tetraselmis* sp. GSL018, *Fistulifera solaris* and *Symbiodinium microadriaticum*) ABCB1/MDR1 transporter proteins.

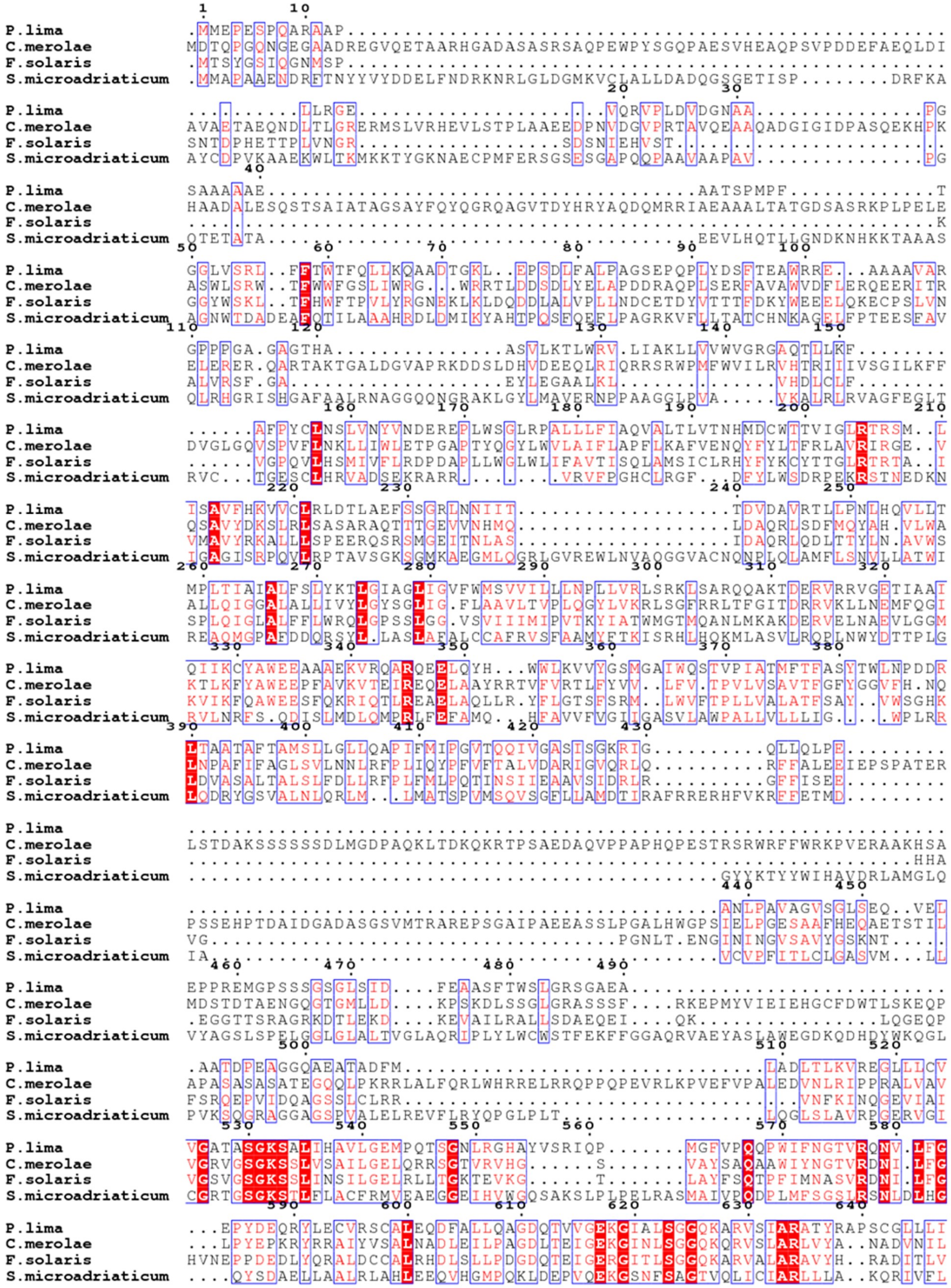
P.lima .MMEPESPQARAAP...
C.merolae MDTQPGQNGEGAADREGVQETAARHGADASASRSAQPEWPYSGQPAESVHEAQPSVPDDEFAEQLDI
F.solaris .MTSYGSIQGNMSP...
S.microadriaticum .MMAPAAENDRFTNYYVYDDELFNDRKNRLGLDGMKVCLALLDADQGSGETISP........DRFKA
P.lima ..........LLRGE....................VQRVPLDVDGNAA...............PG
C.merolae AVAETAEQNDLTLGRERMSLVRHEVLSTPLAAEEDPNVDGVPRTAVQEAAQADGIGIDPASQEKHPK
F.solaris SNTDPHETTPLVNGR...............SDSNIEHVST..........................
S.microadriaticum AYCDPVKAAEKWLTKMKKTYGKNAECPMFERSGSESGAPQQPAAVAAPAV...............PG
P.lima SAAAAAE..................................AATSPMPF.............T
C.merolae HAADALESQSTSAIATAGSAYFQYQGRQAGVTDYHRYAQDQMRRIAEAAALTATGDSASRKPLPELE
F.solaris ....K
S.microadriaticum QTETATA...................................EEVLHQTLLGNDKNHKKTAAAS
P.lima GGLVSRL..FFTWTFQLLKQAADTGKL..EPSDLFALPAGSEPQPLYDSFTEAWRRE...AAAAVAR
C.merolae ASWLSRW..TFWWFGSLIWRG...WRRTLDDSDLYELAPDDRAQPLSERFAVAWVDFLERQEERITR
F.solaris GGYWSKL..TFHWFTPVLYRGNEKLKLDQDDLALVPLLNDCETDYVTTTFDKYWEEELQKECPSLVN
S.microadriaticum AGNWTDADEAFQTILAAAHRDLDMIKYAHTPQSFQEFLPAGRKVFLLTATCHNKAGELFPTEESFAV
P.lima GPPPGA.GAGTHA...............ASVLKTLWRV.LIAKLLVWVGRGAQTLLKF........
C.merolae ELERER.QARTAKTGALDGVAPRKDDSLDHVDEEQLRIQRRSRWPMFWVILRVHTRIIIVSGILKFF
F.solaris ALVRSF.GA...................EYLEGAALKL...............VHDLCLF........
S.microadriaticum QLRHGRISHGAFAALRNAGGQQNGRAKLGYLMAVERNPPAAGGLPVA.....VKALRLRVAGFEGLT
P.lima ......AFPYCLNSLVNYVNDEREPLWSGLRPALLLFIAQVALTLVTNHMDCWTTVIGLRTRSM..L
C.merolae DVGLGQVSPVFLNKLLIWLETPGAPTYQGYLWVLAIFLAPFLKAFVENQYFYLTFRLAVRIRGE..V
F.solaris ......VGPQVLHSMIVFLRDPDAPLLWGLWLIFAVTISQLAMSICLRHYFYKCYTTGLRTRTA..I
S.microadriaticum RVC...TGESCLHRVADSEKRARR..........VRVFPGHCLRGF...DFYLWSDRPEKRSTNEDKN
P.lima ISAVFHKVVCLRLDTLAEFSSGRLNNIIT...................TDVDAVRTLLPNLHQVLLT
C.merolae QSAVYDKSLRLSASARAQTTTGEVVNHMQ...................LDAQRLSDFMQYAH.VLWA
F.solaris VMAVYRKALLLSPEERQSRSMGEITNLAS...................IDAQRLQDLTTYLN.AVWS
S.microadriaticum IGAGISRPQVLRPTAVSGKSGMKAEGMLQGRLGVREWLNVAQGGVACNQNPLQLAMFLSNVLLATWI
P.lima MPLTIAIALFSLYKTLGIAGLIGVFWMSVVILLLNPLLVRLSRKLSARQQAKTDERVRRVGETIAAI
C.merolae ALLQIGGALALLIVYLGYSGLIG.FLAAVLTVPLQGYLVKRLSGFRRLTFGITDRRVKLLNEMFQGI
F.solaris SPLQIGLALFFLWRQLGPSSLGG.VSVIIIMIPVTKYIATWMGTMQANLMKAKDERVELNAEVLGGM
S.microadriaticum REAQMGPAFDDQRSYL.LASLAFALCCAFRVSFAAMYFTKISRHLHQKMLASVLRQPLNWYDTTPLG
P.lima QIIKCYAWEEAAAEKVRQARQEELQYH...WWLKVVYGSMGAIWQSTVPIATMFTFASYTWLNPDDR
C.merolae KTLKFYAWEEPFAVKVTEIREQEELAAYRRTVFVRTLFYVV..LFV.TPVLVSAVTFGFYGGVFH.NQ
F.solaris KVIKFQAWEESFQKRIQTLREAEELAQLLR.YFLGTSFSRM..LWVFTPLLVALATFSAY..VWSGHK
S.microadriaticum RVLNRFS.QDISLMDLQMPRLFEFAMQ...HFAVVFVGLIGASVLAWPALLVLLIG.....WPLRR
P.lima LTAATAFTAMSLLGLLQAPIFMIPGVTQQIVGASISGKRIG..........QLLQLPE.........
C.merolae LNPAFIFAGLSVLNNLRFPLIQYPFVFTALVDARIGVQRLQ..........RFFALEEIEPSPATER
F.solaris LDVASALTALSLFDLLRFPLFMLPQTINSIIEAAVSIDRLR..........GFFISEE.........
S.microadriaticum LQDRYGSVALNLQRLM...LMATSPVMSQVSGFLLAMDTIRAFRRERHFVKRFFETMD.........
P.lima ....
C.merolae LSTDAKSSSSSSDLMGDPAQKLTDKQKRTPSAEDAQVPPAPHQPESTRSRWRFFWRKPVERAAKHSA
F.solaris ....HHA
S.microadriaticum .....................................GYYKTYYWIHAVDRLAMGLQ
P.lima ....ANLPAVAGVSGLSEQ..VEL
C.merolae PSSEHPTDAIDGADASGSVMTRAREPSGAIPAEEASSLPGALHWGPSIELPGESAAFHEQAETSTIL
F.solaris VG.................................PGNLT.ENGINININGVSAVYGSKNT.....
S.microadriaticum IA...VCVPFITLCLGASVM...LL
P.lima EPPREMGPSSSGSGLSID....FEAASFTWSLGRSGAEA..........................
C.merolae MDSTDTAENGQGTGMLLD....KPSKDLSSGLGRASSSF....RKEPMYVIEIEHGCFDWTLSKEQP
F.solaris .EGGTTSRAGRKDTLEKD....KEVAILRALLSDAEQEI....QK..............LQGEQP
S.microadriaticum VYAGSLSPELGGLGLALTVGLAQRIPLYLWCWSTFEKFFGGAQRVAEYASLAWEGDKQDHDYWKQGL
P.lima .AATDPEAGGQAEATADFM...............................LADLTLKVREGLLCV
C.merolae APASASASATEGQQLPKRRLALFQRLWHRRELRRQPPQPEVRLKPVEFVPALEDVNLRIPPRALVAV
F.solaris FSRQEPVIDQAGSSLCLRR...............................VNFKINQGEVIAI
S.microadriaticum PVKSQGRAGGAGSPVALELREVFLRYQPGLPLT.................LQGLSLAVRPGERVGI
P.lima VGATASGKSALIHAVLGEMPQTSGNLRGHAYVSRIQP.......MGFVPQQPWIFNGTVRQNV.LFG
C.merolae VGRVGSGKSSLVSAILGELQRRSGTVRVHG......S........VAYSAQAAWIYNGTVRDNI.LFG
F.solaris VGSVGSGKSSLINSILGELRLLTGKTEVKG......T........LAYFSQTPFIMNASVRDNI.LFG
S.microadriaticum CGRTGSGKSTLFLACFRMVEAEGGEIHVWGQSAKSLPLPELRASMAIVPQDPLMFSGSLRSNLDLHG
P.lima ...EPYDEQRYLECVRSCALEQDFALLQAGDQTVVGEKGIALSGGQKARVSIARATYRAPSCGLLLI
C.merolae ...LPYEPKRYRRAIYVSALNADLEILPAGDLTEIGEKGINLSGGQKQRVSLARLVYA..NADVNIL
F.solaris HVNEPPDEDLYQRALDCCALRHDLSLLPDGDQTEIGERGITLSGGQKARVALARAVYH..RADITLV
S.microadriaticum ....QYSDAELLAALRLAHLEEQVHGMPQKLDEPVQEKCSNFSACTVQLICIARLLLA..KQRIVFL

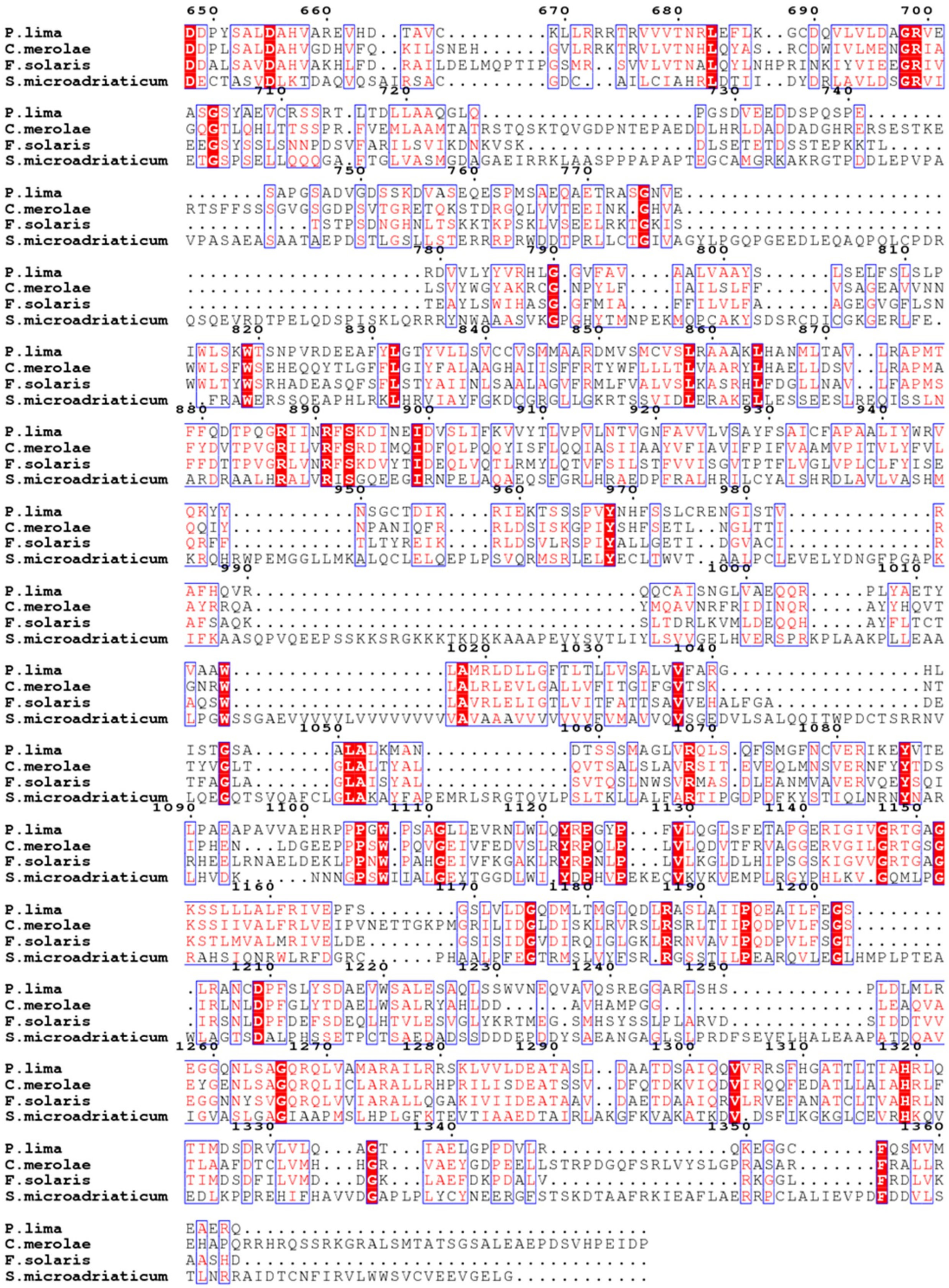

Supplementary Figure 2 Alignment of ABCC1 from *P. lima* with other algae (*Cyanidioschyzon merolae* strain 10D, *Fistulifera solaris*, and *Symbiodinium microadriaticum*) ABCC1/MRP1 transporter proteins.

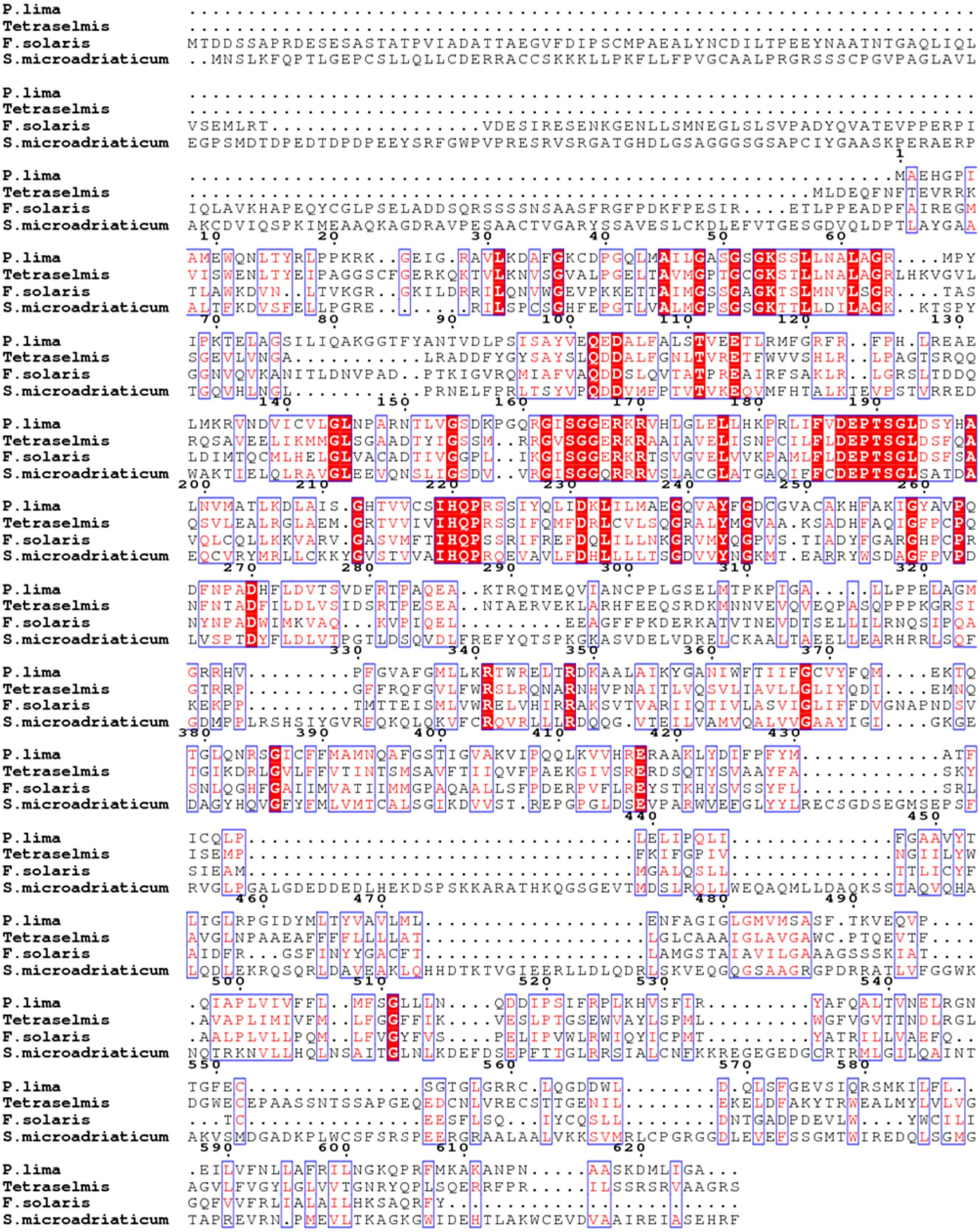

Supplementary Figure 3 Alignment of ABCG2 from *P. lima* with other algae (*Tetraselmis* sp. GSL018, *Fistulifera solaris* and *Symbiodinium microadriaticum*) ABCG2 transporter proteins.

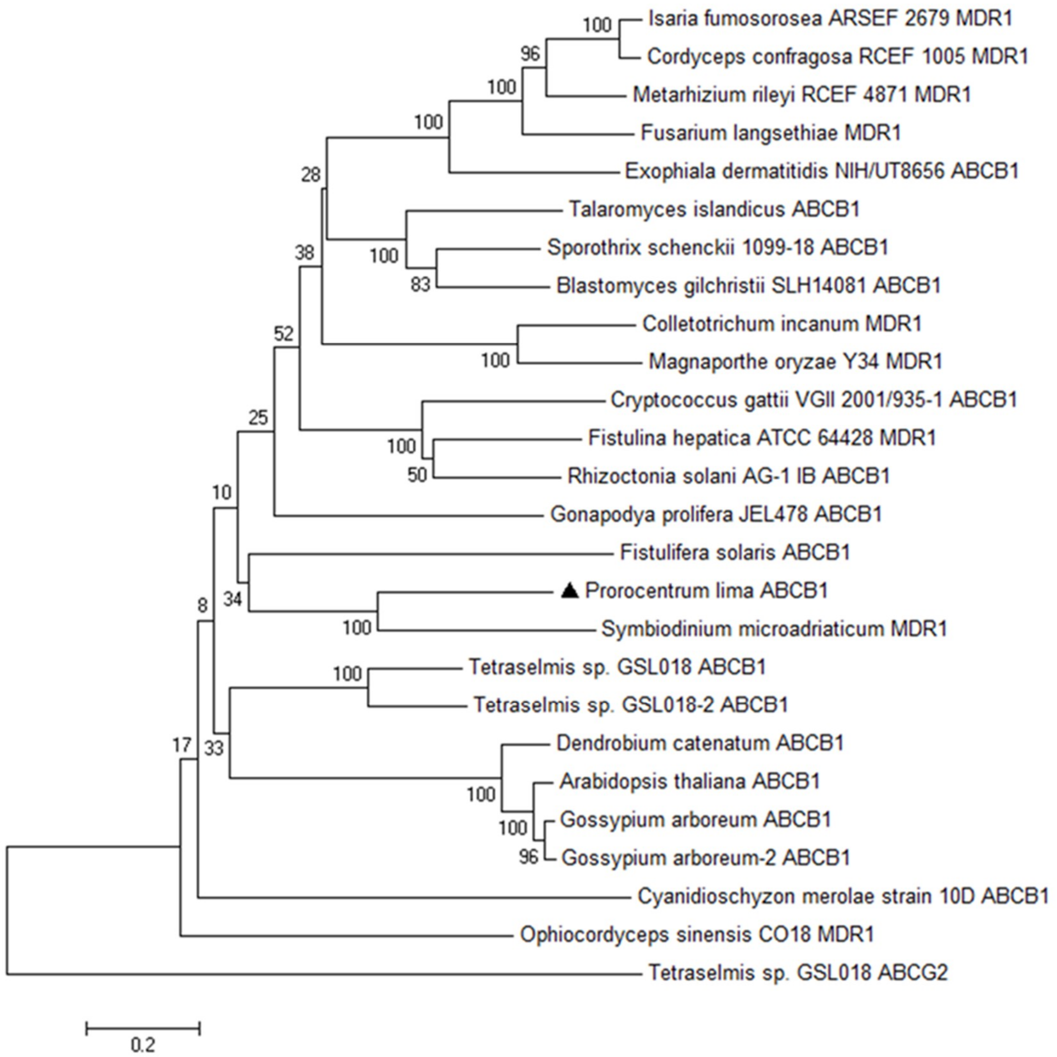

Supplementary Figure 4 Phylogenetic tree based on multiple alignment (Clustal 3.0) of various ABCB1/MDR1 transporter amino acid sequences from various plants and algae. Tree was generated using the neighbor-joining method. Percent concordance shown at the nodes is based on 1000 bootstrap iterations. ABCB1 transporters are as follows: MDR1 (XP_018701169.1) for *Isaria fumosorosea* ARSEF 2679; MDR1 (OAA74823.1) for *Cordyceps confragosa* RCEF 1005; MDR1 (OAA35161.1) for *Metarhizium rileyi* RCEF 4871; MDR1 (KPA41886.1) for *Fusarium langsethiae*; ABCB1 (EHY53853.1) for *Exophiala dermatitidis* NIH/UT8656; MDR1 (CRG91977.1) for *Talaromyces islandicus*; MDR1 (KJR80784.1) for *Sporothrix schenckii* 1099-18; MDR1 (OAT13273.1) for *Blastomyces gilchristii* SLH14081; MDR1 (KZL80199.1) for Colletotrichum incanum; MDR1 (ELQ42255.1) for *Magnaporthe oryzae* Y34; ABCB1 (KIS00281.1) for *Cryptococcus gattii* VGII 2001/935-1; MDR1 (KIY44784.1) for *Fistulina hepatica* ATCC 64428; ABCB1 (CEL54430.1) for *Rhizoctonia solani* AG-1 IB; ABCB1 (KXS15947.1) for *Gonapodya prolifera* JEL478 ; ABCB1 (GAX29293.1) for *Fistulifera solaris* ; MDR1 (OLQ15128.1) for *Symbiodinium microadriaticum*; ABCB1 (JAC83958.1) for *Tetraselmis* sp. GSL018; ABCB1 (JAC73905.1) for *Tetraselmis* sp. GSL018-2; ABCB1 (XP_020679153.1) for *Dendrobium catenatum*; ABCB1 (NP_181228.1) for *Arabidopsis thaliana*; ABCB1 (KHG30028.1) for *Gossypium arboreum*; ABCB1 (KHG25987.1) for *Gossypium arboreum* -2; ABCB1 (XP_005535498.1) for *Cyanidioschyzon merolae* strain 10D; MDR1 (EQL01639.1) for *Ophiocordyceps sinensis* CO18. ABCG2 (JAC83372.1) for *Tetraselmis* sp. GSL018.

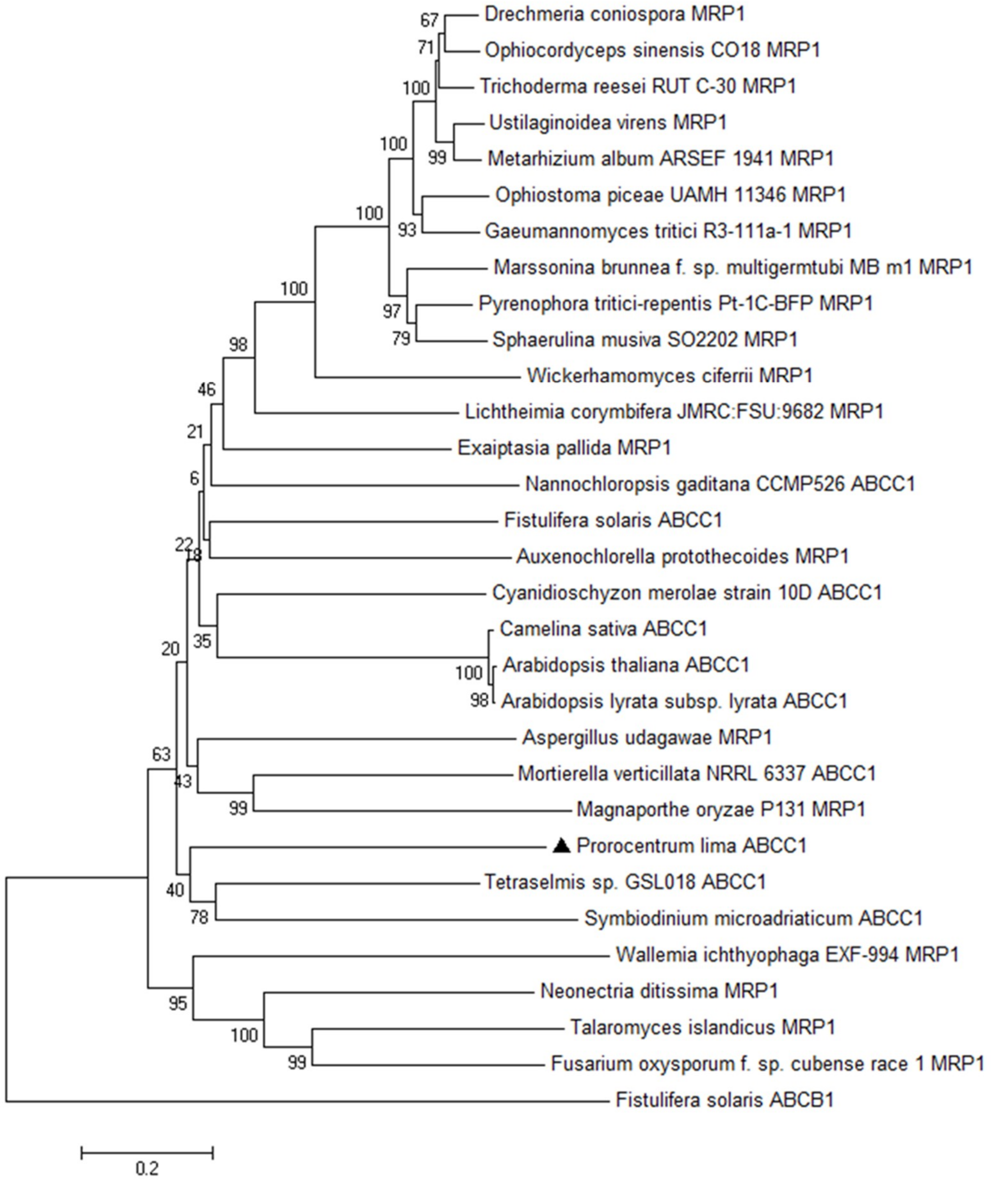

Supplementary Figure 5 Phylogenetic tree based on multiple alignment (Clustal 3.0) of various ABCC1/MRP1 transporter amino acid sequences from various plants and algae. Tree was generated using the neighbor-joining method. Percent concordance shown at the nodes is based on 1000 bootstrap iterations. ABCC1 transporters are as follows: MRP1 (KYK61458.1) for *Drechmeria coniospora*; MRP1 (EQL01282.1) for *Ophiocordyceps sinensis* CO18; MRP1 (ETS00260.1) for *Trichoderma reesei* RUT C-30; MRP1 (KDB17864.1) for *Ustilaginoidea virens*; MRP1 (KHO00668.1) for *Metarhizium album* ARSEF 1941; MRP1 (EPE10310.1) for *Ophiostoma piceae* UAMH 11346; MRP1 (EJT81949.1) for *Gaeumannomyces tritici* R3-111a-1; MRP1 (EKD20282.1) for *Marssonina brunnea* f. sp. 'multigermtubi' MB_m1; MRP1 (EDU40488.1) for *Pyrenophora tritici-repentis* Pt-1C-BFP; MRP1 (EMF11638.1) for *Sphaerulina musiva* SO2202; MRP1 (CCH41464.1) for *Wickerhamomyces ciferrii*; MRP1 (CDH53089.1) for *Lichtheimia corymbifera* JMRC:FSU:9682; MRP1 (XP_020903336.1) for *Exaiptasia pallida*; ABCC1 (EKU20214.1) for *Nannochloropsis gaditana* CCMP526; ABCC1 (GAX21062.1) for *Fistulifera solaris*; MRP1 (KFM23846.1) for *Auxenochlorella protothecoides*; ABCC1 (XP_005537371.1) for *Cyanidioschyzon merolae* strain 10D; ABCC1 (XP_019094624.1) for *Camelina sativa*; MRP1 (NP_001031116.1) for *Arabidopsis thaliana*; ABCC1 (XP_020866423.1) for *Arabidopsis lyrata* subsp. lyrata; MRP1 (GAO82707.1) for *Aspergillus udagawae*; ABCC1 (KFH70890.1) for *Mortierella verticillata* NRRL 6337; MRP1 (ELQ66541.1) for *Magnaporthe oryzae* P131; ABCC1 (JAC66611.1) for *Tetraselmis* sp. GSL018; MRP1 (OLQ15328.1) for *Symbiodinium microadriaticum*; MRP1 (XP_009270082.1) for *Wallemia ichthyophaga* EXF-994 ; MRP1 (KPM36182.1) for *Neonectria ditissima*; MRP1 (CRG89601.1) for *Talaromyces islandicus*; MRP1 (ENH68971.1) for *Fusarium oxysporum* f. sp. cubense race 1. ABCB1 (GAX29293.1) for *Fistulifera solaris*.

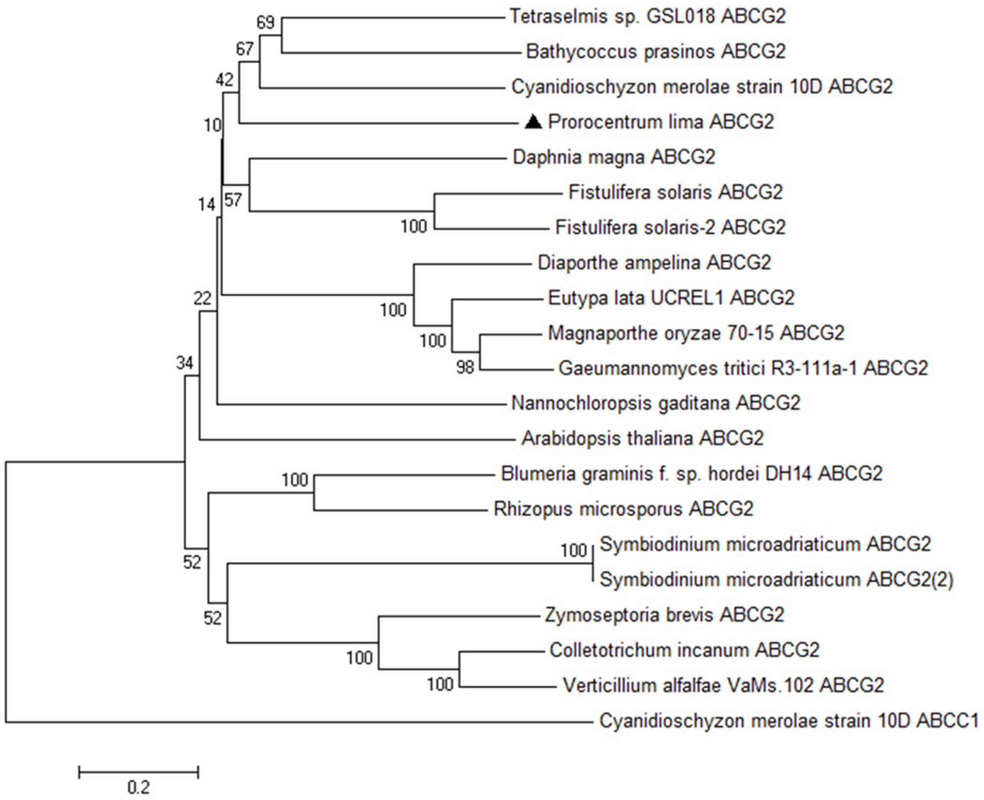

Supplementary Figure 6 Phylogenetic tree based on multiple alignment (Clustal 3.0) of various ABCG2 transporter amino acid sequences from various plants and algae. Tree was generated using the neighbor-joining method. Percent concordance shown at the nodes is based on 1000 bootstrap iterations. ABCG2 transporters are as follows: ABCG2 (JAC83372.1) for *Tetraselmis* sp. GSL018; ABCG2 (XP_007509928.1) for *Bathycoccus prasinos*; ABCG2 (BAM82508.1) for *Cyanidioschyzon merolae* strain 10D; ABCG2 (KZS09610.1) for *Daphnia magna*; ABCG2 (GAX11407.1) for *Fistulifera solaris*; ABCG2 (GAX12580.1) for *Fistulifera solaris*-2; ABCG2 (KKY38206.1) for *Diaporthe ampelina*; ABCG2 (EMR71650.1) for *Eutypa lata* UCREL1; ABCG2 (XP_003713947.1) for *Magnaporthe oryzae* 70-15; ABCG2 (EJT81949.1) for *Gaeumannomyces tritici* R3-111a-1; ABCG2 (EWM27479.1) for *Nannochloropsis gaditana*; ABCG2 (OAP09062.1) for *Arabidopsis thaliana*; ABCG2 (CCU81831.1) for *Blumeria graminis* f. sp. hordei DH14; ABCG2 (CEG64621.1) for *Rhizopus microsporus*; ABCG2 (OLQ08707.1) for *Symbiodinium microadriaticum*; ABCG2 (OLP84914.1) for *Symbiodinium microadriaticum*; ABCG2 (KJX95289.1) for *Zymoseptoria brevis*; ABCG2 (OHW95109.1) for *Colletotrichum incanum*; ABCG2 (XP_003007046.1) for *Verticillium alfalfae* VaMs.102. ABCC1 (XP_005537371.1) for *Cyanidioschyzon merolae* strain 10D.
